# Customisable Tablet Printing: The Development of Multimaterial Hot Melt Inkjet 3D Printing to Produce Complex and Personalised Dosage Forms

**DOI:** 10.3390/pharmaceutics13101679

**Published:** 2021-10-14

**Authors:** Anna Lion, Ricky D. Wildman, Morgan R. Alexander, Clive J. Roberts

**Affiliations:** 1 Division of Advanced Materials and Healthcare Technologies, School of Pharmacy, The University of Nottingham, Nottingham NG7 2RD, UK; morgan.alexander@nottingham.ac.uk; 2 Centre for Additive Manufacturing, Faculty of Engineering, University of Nottingham, Nottingham NG7 2RD, UK; ricky.wildman@nottingham.ac.uk

**Keywords:** 3D printing, hotmelt inkjet printing, solid dosage form, polypill, multi-material tablets, drug delivery

## Abstract

One of the most striking characteristics of 3D printing is its capability to produce multi-material objects with complex geometry. In pharmaceutics this translates to the possibility of dosage forms with multi-drug loading, tailored dosing and release. We have developed a novel dual material hot-melt inkjet 3D printing system which allows for precisely controlled multi-material solvent free inkjet printing. This reduces the need for time-consuming exchanges of printable inks and expensive post processing steps. With this printer, we show the potential for design of printed dosage forms for tailored drug release, including single and multi-material complex 3D patterns with defined localised drug loading where a drug-free ink is used as a release-retarding barrier. For this, we used Compritol HD5 ATO (matrix material) and Fenofibrate (model drug) to prepare both drug-free and drug-loaded inks with drug concentrations varying between 5% and 30% (*w*/*w*). The printed constructs demonstrated the required physical properties and displayed immediate, extended, delayed and pulsatile drug release depending on drug localisation inside of the printed formulations. For the first time, this paper demonstrates that a commonly used pharmaceutical lipid, Compritol HD5 ATO, can be printed via hot-melt inkjet printing as single ink material, or in combination with a drug, without the need for additional solvents. Concurrently, this paper demonstrates the capabilities of dual material hot-melt inkjet 3D printing system to produce multi-material personalised solid dosage forms.

## 1. Introduction

Humans are living longer today than ever before, and as a result we are seeing a significant increase in the number of patients suffering from multiple chronic diseases. Currently, the primary approach to treat such complex cases is polypharmacy, a broad term that includes drug administration regimens in which patients are required to take multiple medicines several times a day [1,2,3,4,5,6]. While this approach is effective, it can lead to low compliance (32.6%) among older patients [6]. Such data are linked not only to the complexity of the therapy, which reduces patient compliance and adherence, but also to the “one drug, one dose fits all” approach necessarily adopted by most physicians [7].

A potential solution is the use of personalised medicines tailored to the individual patient. Traditionally, for solid dosage forms this has been achieved by controlling the release profile using barrier layers, and by manipulating the excipient’s physical and chemical properties [8,9,10,11]. However, these processes are highly complex, and the performance is limited, since only a small variety of outcomes can be achieved.

Three-dimensional printing offers the potential for an economical, sustainable and customisable manufacturing approach for personalised tablets for individual patient needs. In recent decades, 3D printing has been recognised by many to be an answer to this call and the future of polypharmacy [12,13,14,15,16,17,18,19,20,21,22,23,24,25,26,27,28,29]. The possibility to mass-produce personalised components without substantially increasing production time or costs makes 3D printing an attractive proposition [12,13,14]. This type of manufacturing becomes particularly beneficial in the treatment of patients in paediatric age or with swallowing issues. Specifically, it is possible to produce tablets with small diameter between 1.5 and 4.0 mm [30] or with appealing shape and texture [31].

Here, we propose the adaptation of a commercially available 3D hot-melt inkjet printer (PiXDRO LP50) for the manufacture of multi-material and tuneable pharmaceutical dosage forms. We aimed to develop a double unit system capable of printing two different materials during the same additive process and to exploit a depositional resolution of up to 50 µm to produce tailored drug release profiles. The method therefore not only allows control over the macro-structure, but also the local microstructure of the tablet.

The use of a hot-melt jetting approach rather than a standard solvent-based inkjet printer removes the need for a solvent in the formulation, reducing potential issues related to residual solvent and solvent mediated stability issues and provides a ready means of forming the solid form on cooling. The ink is stored in a reservoir heated above its melting point, and no solvent is thus required to keep the ink in its liquid form. Here, we used FDA-approved Compritol HD5 ATO as the carrier and Fenofibrate as the model drug.

Investigation of the thermal and mechanical properties alongside drug distribution was performed before and after printing to ensure consistency with the intended computational design and that no drug degradation occurred during printing. The influence of dosage form surface area on drug release profile was investigated by printing ‘honeycomb geometry’ tablets with increasingly small cell size until a solid tablet was formed [32]. We also designed and prepared tablets with delayed and pulsatile release by printing drug-free barrier layers, inside the tablet to delay the moment of interaction between the drug-loaded layers and the dissolution media.

## 2. Materials and Methods

### 2.1. Materials

Compritol HD5 ATO (behenoyl polyoxyl-8 glycerides) was kindly donated by Gattefosse’ (Saint-Priest, France), and Fenofibrate (2-[4-(4-Chlorobenzoyl) phenoxy]-2-methylpropanoic acid isopropyl ester) >99% powder was purchased from Sigma Aldrich (Darmstadt, Germany). The salts for the dissolution media, namely Sodium Dodecil Sulphate, Sodium Phosphate Monobasic Monohydrate and Sodium Phosphate Dibasic Anhydrate were purchased from Sigma Aldrich (Darmstadt, Germany). All materials were used as received.

### 2.2. Printable Ink Preparation

Ink mixtures were obtained by adding a suitable quantity of Fenofibrate to the melted matrix material until the desired concentration was reached, namely 5%, 10%, 20% or 30% *w*/*w*. The mixture was then left to stir for 30–40 min on a hotplate at 100 °C, allowing the drug to melt completely and form a homogeneous mixture with the wax. The resulting ink was then transferred into one of the two reservoirs attached to the printer ready to be used. The drug-free ink was prepared by loading the Compritol HD5 ATO directly into the printer’s other reservoir and was melted in situ.

### 2.3. Tablets Fabrication and 3D Printing System

**Figure 1:**
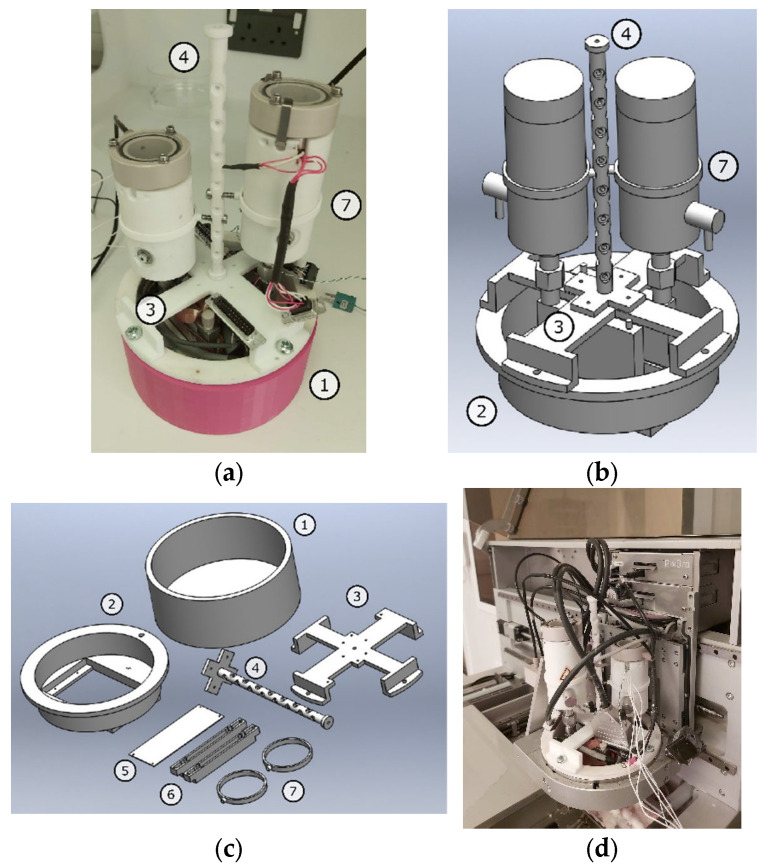
(**a**) Photo and (**b**) rendered image of the assembled prototype. (**c**) Rendered image displaying of the main supports parts: assembly resting holder (1), base support (2), top case (3), central anchoring structure (4), bottom cover (5), printhead holder (6), reservoir holder (7). (**d**) Close-up of the dual-head printing unit mounted in the LP50 printer.

We used a commercially available inkjet printer (PiXDRO LP50, Meyer Burger, Gwatt, Switzerland), originally designed to hot-melt print mono-material objects. To allow the fabrication of printlets with multi-material complex 3D patterns with defined localised composition, an in-house built assembly containing two hot-melt units was designed and developed capable of delivering different materials on demand. Each one of the two hot-melt units was composed of a heated reservoir rigidly connected to a piezoelectric activated printing head equipped with a heating element (Figure 1). The print head of choice was a Spectra SE-128 AA (Fujifilm Dimatix, Santa Clara, CA, USA) which has 128 nozzles, 35 µm in diameter with a 508 µm spacing. Such a system produces droplets of approximately 30 picolitres in volume, this value is provided by the manufacturer and depends upon the nozzle geometry and working conditions.

An additional temperature control system was implemented for the second hot-melt print unit. A revised control software implementation was also necessary to account for the new setup.

During the printing, the reservoir and printing head temperature were set at 90 °C and the firing voltage to 100 V. The printing conditions were selected to comply with the material thermo-mechanical properties and ensure optimal printability. Printing was done onto polyethylene terephthalate (PET) substrates kept at a constant temperature of 30 °C.

### 2.4. Tablet Designs

Following FDA guidelines [33] the Immediate and Sustained release tablets’ default shape for this study was cylindrical. Sustained release tablets had a designed diameter of 10 mm and a target weight of 350 mg. Tablets with increasing drug content were prepared, namely 5%, 10%, 20% and 30% *w*/*w*.

Immediate (more rapid) drug delivery tablets were designed with an increased surface area using the same formulation and a honeycomb internal geometry, following the approaches demonstrated by Kyobula, Khaled and Goyanes [24,27,32]. A modelling computer-aided software (SolidWorks) was utilised to design the honeycomb geometries with varying channel diameters, specifically achieving a 45% infill ⌀_cell_ = 0.60 mm, 24% infill ⌀_cell_ = 1.22 mm and 17% infill ⌀_cell_ = 1.83 mm.

Tablets with a constant drug loading (10% *w*/*w*) and a varying infill pattern were prepared with overall diameters of (10.04 ± 0.04) mm, and heights (thickness) of (4.95 ± 0.01) mm. To further explore the effect of tablet geometry on drug release, four more sets of printlets were prepared maintaining the geometry at a constant cell diameter 0.60 mm while increasing drug loading as follows 5%, 10%, 20% and 30% *w*/*w*.

Delayed and pulsatile release tablets were designed to display specific times of drug release exploiting the multi material jetting system and produce a precisely controlled local drug distribution within the tablet.

For the delayed release tablets, the final geometry included a drug loaded centre surrounded by a drug free shell acting as a rate-limiting barrier to drug release. To understand the effect of shell thickness on the dissolution drug release profile, the core was identical in all preparations (⌀ = 8 mm, *h* = 2 mm and 10% *w*/*w* drug loading) while the shell thickness was varied as follows *s*_1_ = 1 mm, *s*_2_ = 1.5 mm and *s*_3_ = 2 mm. The choice of external layer thickness was designed to keep the tablet size within FDA recommended dimensions [34]. A second type of tablet was designed to explore the effect of drug concentration on drug release with a fixed shell thickness. In this case, the tablet geometry was kept unchanged (core: ⌀ = 8 mm, *h* = 2 mm, shell: *s* = 1 mm) while the drug concentration varied between 5% and 30% *w*/*w*.

Lastly, a multi-compartment dosage form strategy was chosen to investigate pulsatile release [35]. In this case, the tablet contained two nested drug-loaded regions encapsulated by drug-free barrier material. A drug loaded central disc region (⌀ = 4 mm, *h* = 2 mm) was surrounded by a 3 mm shell of drug free material that was furthermore encased by a concentric ring (*s* = 1 mm, *h* = 7 mm), with a final 1 mm layer of drug free material encasing the entire object. The final tablet dimensions were designed to be ⌀ = 10 mm and *h* = 9 mm.

### 2.5. Differential Scanning Calorimetry (DSC)

DSC measurements were carried out using a DSC 8000 (Perkin Elmer, Waltham, MA, USA). All samples were weighed (between 5 and 10 mg) using a high precision scale (Kern ALS Analytical Balance, Balingen, Germany) and encased in hermetically sealed aluminium pans (TA standard aluminium sample pans and lids) to retain the volatile components. Samples were heated at a rate of 10 °C min^−1^ in a nitrogen enriched environment at a steady flow rate of 20 mL min^−1^.

### 2.6. Thermogravimetric Analysis (TGA)

A TGA 4000 thermogravimetric analyser (Perkin Elmer, Waltham, MA, USA) was used to study materials degradation, 10 mg to 15 mg of sample was placed into the instrument pot and a scan was run between 30 and 700 °C at 20 °C min^−1^. For both techniques, data collection was performed using TA Advantage software.

### 2.7. Powder X-ray Diffraction (pXRD)

pXRD analysis was performed using an XRD D8 Advance DaVinci (Bruker, Billerica, MA, USA) using Cu Kα radiation (40 kV and 40 mA) and an Ni filter with a scanning speed of 0.02° 2θ s^−1^ with the divergence slit set to 0.3°. The anti-scatter slit was set at 5.2 mm, which is fully open, as required for fast scanning with the detector in 1D mode. Spectra were collected at room temperature and in the 3 to 70 2θ range.

Samples were prepared by crushing the printed tables utilising a mortar and pestle into a fine powder, which was then transferred into a shallow PMMA sample holder (8.5 mm height and 25 mm diameter) from Bruker (Billerica, MA, USA). Once loaded, the sample was pressed and the top surface smoothed using a glass slide, sample was added until a smooth surface was achieved. Any loose material present on the rim of the disc holder was removed using an isopropanol-imbued tissue. All data analysis was performed using OriginPro 9.0.

### 2.8. Scanning Electron Microscopy (SEM) and Energy-Dispersive X-ray (EDX)

SEM and EDX images were obtained using a variable pressure scanning electron microscope (TM3030 Hitachi, Tokyo, Japan) equipped with a multi axis stage controller (Debem Sprite) in a high vacuum at an accelerated voltage ranging between 10 and 20 kV depending on the specific sample. Each sample was prepared by mounting section of the original tablet onto an aluminium stub (Agar Scientific, Stansted, UK) lined with carbon discs (Agar Scientific, Stansted, UK). Using a Sputter Coater (108 Manual Sputter Coater, Ted Pella, ink., Redding, CA, USA) the samples for SEM analysis, not EDX, were coated with gold for 90 s with a current of 26 mA to 29 mA in a 0.06 mbar Argon environment.

### 2.9. Attenuated Total Reflection-Fourier Transform Infrared Spectroscopy (ATR-FTIR)

ATR-FTIR was used to study drug content inside the different tablets regions to ensure conformity with the intended digital design [36,37]. The analysis was performed using an FTIR Routine Spectrometers Alpha (Bruker, Billerica, MA, USA) equipped with an Attenuated Total Reflection (ATR) crystal. The range of interest was limited to a range of 4000 cm^−1^ to 450 cm^−1^ wavenumber region. The 765 cm^−1^ C–Cl stretch peak was used as a reference to verify the presence of the drug. Three sets of measurements were performed for each region of interest, the results were then analysed and averaged using OriginPro.

### 2.10. Raman Spectroscopy

Raman spectroscopy was used to determine the API content and distribution within the printed solid dosage forms. A HORIBA Jobin Yvon LabRAM HR spectrometer (Kyoto, Japan) was used. The tablets were printed with the surface to be analysed facing a PET substrate and built up from there, to eliminate the ridges characteristic of 3D printing and provide a smooth surface for the analysis. In such a manner subsequent physical cross-sectioning can be avoided eliminating the risk of sample breakages and smearing of materials into neighbouring regions. The samples were mounted on glass slides for Raman analysis. The 1598 cm^−1^ peak, specific to the Fenofibrate, was established as a marker to identify the presence of the drug [38]. All spectra were collected using a 785 nm laser at 25 mW power, a 50× objective, a 300 µm confocal pinhole and a 300 lines/mm diffraction grating. Single spectra acquisition was performed in the range between 100 and 3300 cm^−1^. Data were collected for 5 s with 16 accumulations per spectral window. Mapping was carried out with the same acquisition conditions on an 80 µm line at 10 µm intervals for a total of 9 spectra. The analysis line was located such that the zero of the x-axis fell on the interface line separating the core and shell region. All data were analysed using OriginPro.

### 2.11. In Vitro Release Study

Dissolution studies were performed with a Copley’s Dissolution Test Dis8000 (Nottingham, UK). Samples were sunk in 500 mL of 0.1 M phosphate with 0.05 M sodium lauryl sulphate buffer (pH 7.40 ± 0.05) kept at a constant temperature of 37.0 ± 0.1 °C and constantly stirred at 50 rpm. Then, 5 mL of the sample solution was withdrawn at regular intervals and filtered via 0.45 µm MF-millipore membrane filter (MILLEX HA). An equivalent amount of fresh buffer kept at the same temperature was reintroduced. All dissolution tests were done in triplicate. Pulsatile release tablets represent the only exception to this method; in this case, the samples were collected every 15 min for the entire duration of the experiment and the buffer solution (125 mL) was completely removed and substituted with a fresh one after every sampling session. This again was performed in triplicate.

The amount of drug released into the medium as a function of time was obtained by analysing the collected samples using UV-visible spectrophotometry, a Spark Microplate Reader (Tecan Trading AG Männedorf, Switzerland) UV/Vis Spectrophotometer equipped with a deuterium—tungsten light source. The *λ_max_* = 290 nm peak of maximum absorbance for Fenofibrate was thus used as a reference in the analysis of all samples. All results were analysed using OriginPro.

## 3. Results and Discussion

### 3.1. Thermogravimetric Analysis (TGA)

**Figure 2:**
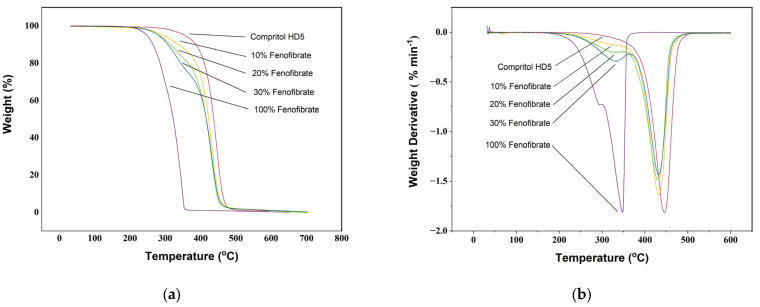
Thermogravimetric curves (**a**) and their derivatives (**b**) of pure Fenofibrate, Compritol HD5 ATO and mixture of the two at 5% (*w*/*w*), 10% (*w*/*w*), 20% (*w*/*w*) and 30% (*w*/*w*).

From the analysis of the TGA curves (Figure 2) of Compritol HD5, Fenofibrate and their printed mixtures the degradation of both materials is complete (>99%) before 500 °C, as is consistent with their organic nature [39,40,41,42,43,44,45]. The TGA curve (Figure 2a) of Compritol HD5 ATO shows a sharp decline in weight starting at 408 °C and terminating at 466 °C. The analysis of the derivative curve (Figure 2b) confirmed the presence of a single thermo-oxidative decomposition with T_d_ = 445 °C. Fenofibrate weight loss was observed over a broader range of temperatures with an onset of 255°C and outset of 353 °C. From the analysis of both TG and its derivative Fenofibrate degradation happens in two different stages with decomposition peak temperatures of 294 and 348 °C. The TGA curves for drug-loaded inks are a direct combination of the two base materials, with the onset temperature lowering with increasing amounts of Fenofibrate.

### 3.2. Differential Scanning Calorimetry (DSC)

**Figure 3:**
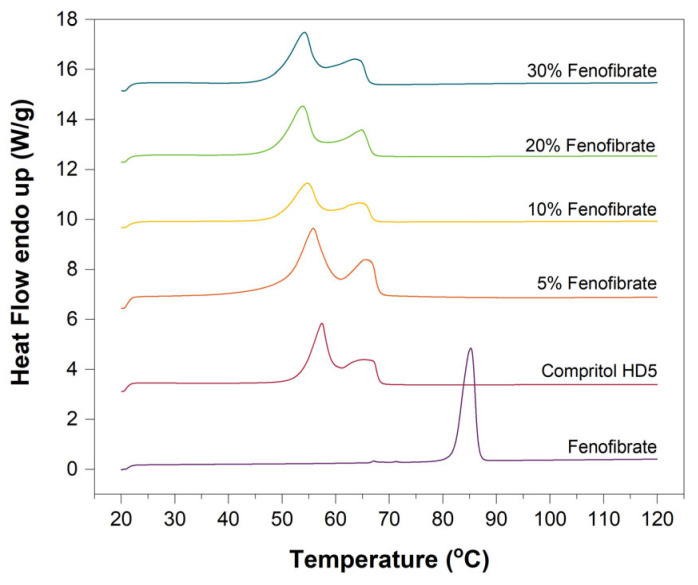
DSC thermograms of pure Fenofibrate and Compritol HD5 alongside with the ink mixture with increasing drug load (5%, 10%, 20% and 30% *w*/*w*).

Figure 3 shows DSC thermograms for Fenofibrate, Compritol HD5 ATO, and their printed mixtures with 5%, 10%, 20% and 30% *w*/*w* Fenofibrate. DSC of Fenofibrate showed one sharp endothermic event at 84.7 ± 0.2 °C consistent with the melting point for Form I [32]. The Compritol HD5 ATO thermogram is characterised by the presence of two endothermic peaks at 57.4 ± 0.1 °C and 66.1 ± 0.3 °C, consistent with the crystalline nature of the two lipid families constituting the material. Namely, Compritol HD5 ATO consists of mono, di-and triglycerides and PEG-8 mono-and di-esters of (C22) behenic acid [46,47].

It should be noted that even at the higher drug concentrations, the DSC thermograms of the fenofibrate-loaded ink compounds did not show a melting endotherm of the drug and minimal variation in the position of endothermic peaks associated with the carrier. This suggests that the drug remains largely in an amorphous/disordered state, with any ordered component, if present, below the detection limit of DSC [48].

### 3.3. Powder X-ray Diffraction (pXRD)

**Figure 4:**
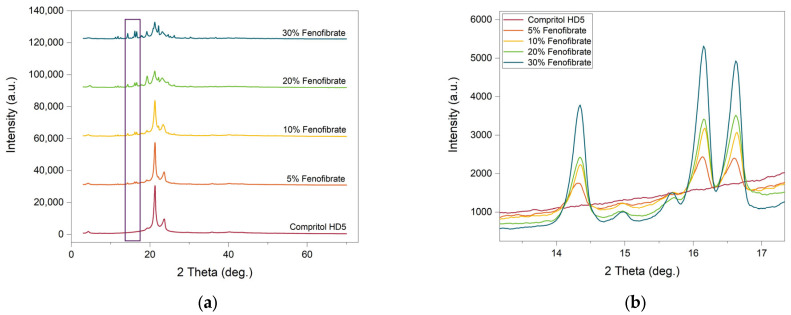
X-ray powder diffraction curves. (**a**) Mixture of the two at 5% (*w*/*w*), 10% (*w*/*w*), 20% (*w*/*w*) and 30% (*w*/*w*). (**b**) Region of interest corresponding to the Fenofibrate characteristic peaks.

pXRD scans of Compritol HD5 ATO and Fenofibrate reveal the presence of diffraction bands characteristic of crystalline substances. Consistent with the DSC analysis where the carrier, Compritol HD5 ATO, showed multiple sharp melting points, the comparable pXRD scan also displayed multiple high-intensity peaks, suggesting that it is a crystalline material. Similar reasoning can be used for pure Fenofibrate as a crystalline material [37]. The diffraction pattern of the ink mixtures (Figure 4) exhibits diffraction peaks which are consistent with the direct combination of the diffractograms of the original components. This suggests that the drug was not completely in a disordered state in the lipidic carrier and remains at least partially in a crystalline state at all weight ratios. These results, combined with the DSC analysis, suggest that only a minor portion, lower than the sensitivity level of the DSC (ca. 5% volume), but more than the sensitivity limit of XRD (ca. 1%) [49] of the Fenofibrate was in a crystalline state. As visible in Figure 4b, higher drug loading corresponds to higher intensity peaks and thus a proportionally greater quantity of Fenofibrate in its crystalline state.

### 3.4. Printed Tablets

**Figure 5:**
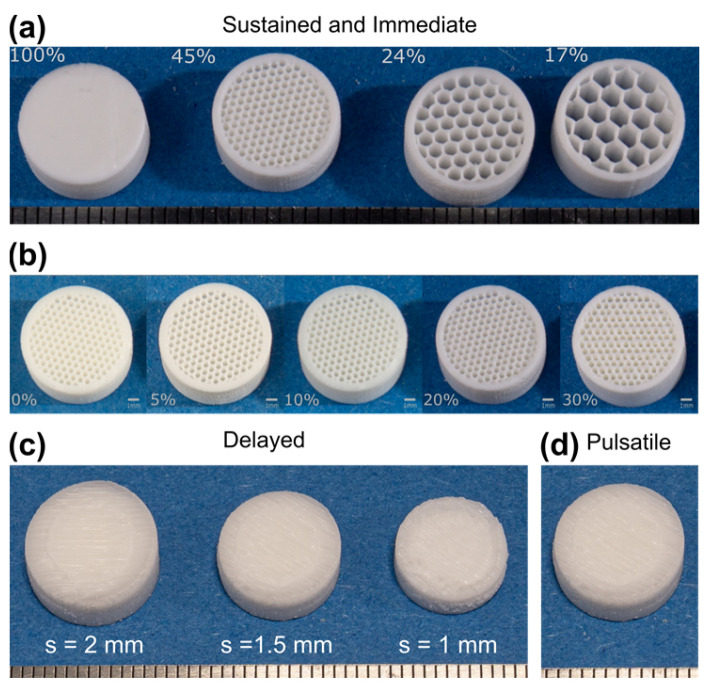
Images of sustained and immediate release tablet (**a**) with constant drug content (10% drug loading) and varying infill, (**b**) with varying drug content (5–30% drug loading) and constant infill (45%). (**c**) Delayed release tablet with 1, 1.5 and 2 mm shell thickness (10% drug loaded core), and (**d**) pulsatile release tablets. Ruler unit: 1 mm.

By visual inspection, all tablets are well defined structurally. In Figure 5, it is possible to appreciate that both the immediate and sustained release tablets present a smooth surface and are without appreciable defects, regardless of the printing infill used or drug content, suggesting that neither of these factors influence the printed outcomes in this aspect.

Similar results can also be seen in the delayed and pulsatile release tablets where the top surface was also smooth except for a small indentation in correspondence of the outside edge of the drug-loaded outermost region, suggesting that the core/shell transition may not be perfectly smooth. Even so, the tablets were solid, well-defined and it was possible to consistently print all three shell size geometries, namely the 1, 1.5 and 2 mm shells. In the case of pulsatile tablets, from a visual inspection no defects are visible in correspondence to the inner core. The resulting tablets were solid and well-defined. It is clear that the printing technique was successful in this regard, and that the printed product does not differ greatly from the intended design.

### 3.5. Scanning Electron Microscopy (SEM) and Energy-Dispersive X-ray (EDX)

**Figure 6:**
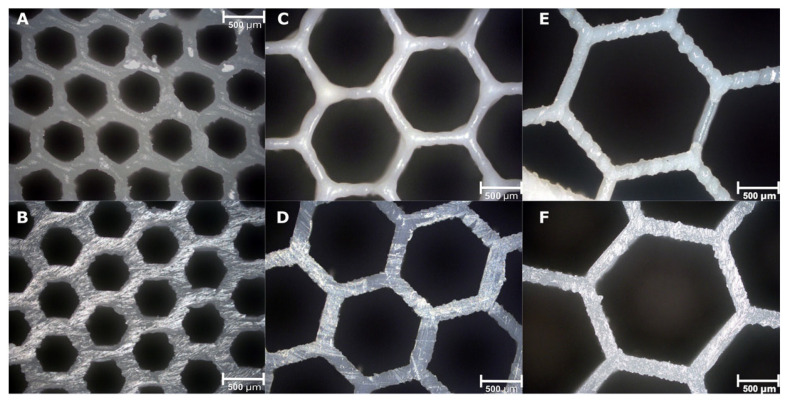
SEM images of 10% drug loading immediate release tablet with channel diameter of (**A**,**B**) 0.6 mm, (**C**,**D**) 1.22 mm and (**E**,**F**) 1.83 mm. (**A**,**C**,**E**) Top surface. (**B**,**D**,**F**) Bottom surface.

From the analysis of SEM images of the infill honeycomb structure (Figure 6), both the channels and the walls are well-formed and, for the most part, free from imperfections.

**Figure 7:**
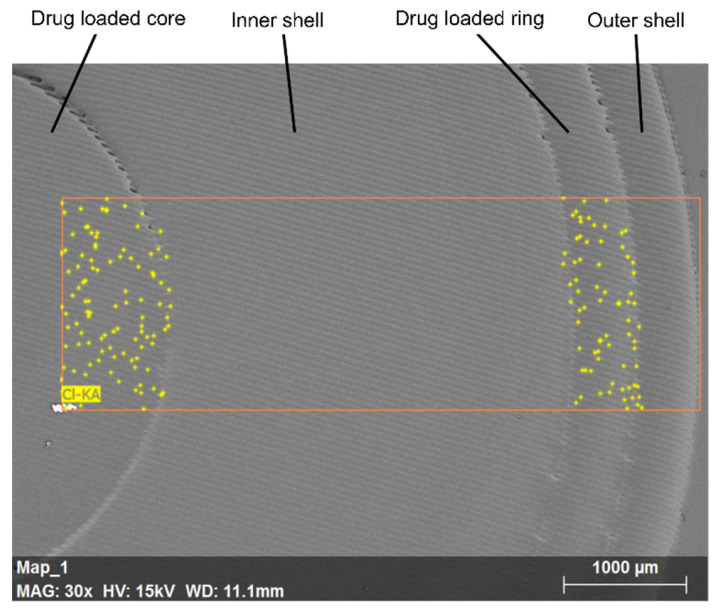
EDX overlap on SEM image of pulsatile release tablets showing localised drug concentration.

Further SEM and EDX examination of the pulsatile release tablets’ internal structure revealed physical cross sectioning (Figure 7); we were thus able to confirm that the print was well-formed, smooth with only minor printing lines observable and only small defects in the top section of the junction line. EDX analysis provided an insight on drug distribution inside of the printlet. The yellow dots in Figure 7 represent signals from chlorine related to the drug (as the only component with chlorine in its structure) showing it to be localised inside the core and ring regions and hence there is no evidence of drug migration. These results are in good agreement with the theoretical design described in Section 2.4, which involved the inclusion of two drug-loaded regions (core and ring) within a drug-free casing which acts as barrier material.

### 3.6. Attenuated Total Reflection-Fourier Transform Infrared Spectroscopy (ATR-FTIR)

**Figure 8:**
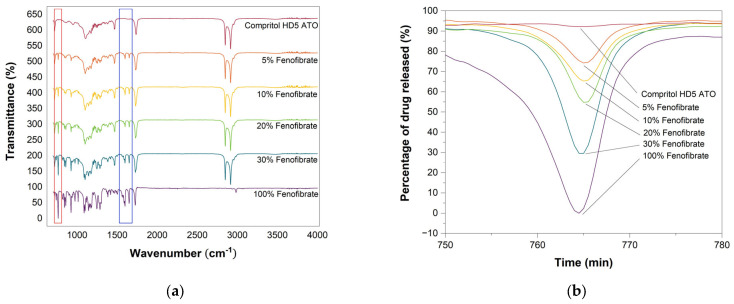
Fourier transform infrared spectroscopy analysis of sustained release tablets of Compritol HD5 ATO and Fenofibrate mixture. (**a**) constant geometry and different drug loading concentration namely 5%, 10%, 20% and 30%. (**b**) Highlight of the 765 cm^−1^ C–Cl peak linked to Fenofibrate presence.

FTIR analysis provided an initial qualitative analysis of the drug content, by identifying the C–Cl bond characteristic of the Fenofibrate molecule within the tablets. By comparison with reference spectra of Fenofibrate concentration it was possible to estimate the content of the API in the printed tablets [50]. In Figure 8a, the peak corresponding to the C–Cl bond are highlighted, with the 765 cm^−1^ C–Cl peak in red and the 1600–1650 cm^−1^ C=C peaks linked to Fenofibrate’s presence in blue.

Figure 8 shows how the sustained release tablets with varied drug content present higher peaks in correspondence to the higher concentrations of Fenofibrate and the peaks intensities are consistent with the expected values as highlighted in Table 1. Similarly, when analysing immediate release tablets with constant geometry, but varied drug content or with varying infill, but constant drug loading, the results are in agreement with each other and with the reference value.

For the investigation of delayed release tablets, samples were separately collected from the core and shell region. The drug was contained only inside the core, and it was absent in the external layers, consistent with the EDX analysis. Notably, for all results, the peaks intensities are in good agreement with the expected values regardless of geometry and drug loading, confirming that not only we were capable of producing tablets with localised drug, but also to successfully tailor the drug concentration.

Lastly, samples from pulsatile release dosage forms were collected from the core, ring and shell region, and the results show that only the spectra relative to the core and ring regions present the peaks related to the drug. Once more, by comparison with the values observed in the previous analysis, the drug concentration is consistent with the estimated 10% loading (Table 1).

Although qualitative, this analysis strengthens the validity of the previous assumptions on the amount of drug loaded into each tablet.

### 3.7. Raman Spectroscopy

**Figure 9:**
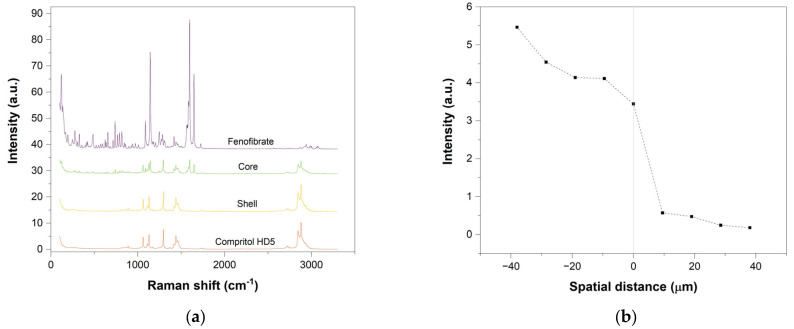
Raman spectra and linear mapping of the core shell interface in Delayed release tablets. (**a**) Spectra of reference Compritol HD5 ATO, printed shell far from the interface, printed core (10% Fenofibrate *w*/*w*) far from the interface and reference Fenofibrate. (**b**) Raman linear map of the chemical characterisation of the core shell interface using the 1598 cm^−1^ peak, unique to the Fenofibrate. Where the x-axis “zero” corresponds to the interface, the negative numbers move into the core region and the positive into the shell section.

Raman spectroscopy was used to better evaluate the spatial distribution of the API inside the multi-material tablets, specifically at the interface between the drug-loaded and drug-free regions. For this purpose, line scans were acquired to produce a chemical characterisation across the core shell interfaces. The 1598 cm^−1^ peak, unique to the Fenofibrate as visible in Figure 9a, was established as a marker to identify the presence of the drug [37].

The analysis line was positioned astride the border between the drug-loaded and drug-free region. As a consequence, the “zero” value of the x-axis was made to correspond with the interface, the negative numbers with the drug-loaded and the positive with drug-free regions.

Figure 9 refers to the Delayed release tablet, graph (**a**) shows the spectra of reference Compritol HD5 ATO, printed shell and printed core (10% Fenofibrate *w*/*w*) far from the interface and reference Fenofibrate, while graph (**b**) represents the linear map of the Fenofibrate characterisation of the core shell interface using the 1598 cm^−1^ peak, unique to Fenofibrate.

All cases show that a clear change in signal occurs when crossing the zero of the system. Meaning that the Fenofibrate is localised in the area corresponding to negative values of the x-axis which identifies the core regions. Nevertheless, minor traces of the API can be found also in the shell region suggesting the possibility of some minor chemical diffusion of Fenofibrate from the main areas of interest to the adjacent shell. Such distribution is furthermore confirmed when analysing region of the sample further away from the interface.

A similar response can be seen when analysing pulsatile release tablets. The results of the two-line maps corresponding to the intersections between core/inner shell and the ring/outside shell once again show a clear change in behaviour when crossing the zero of the system, meaning that the Fenofibrate is concentrated in the area corresponding to negative values of the x-axis which identifies the core and ring regions.

From the analysis of the individual spectra, the characteristic Fenofibrate 1598 cm^−1^ to 1650 cm^−1^ peaks can be found only in the core and shell regions, suggesting drug localisation.

### 3.8. In Vitro Drug Release

**Figure 10:**
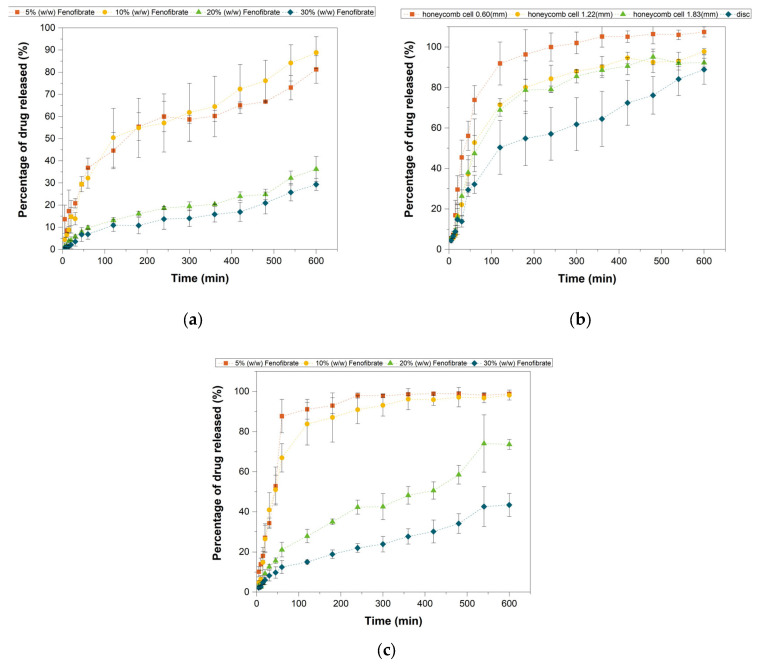
In vitro dissolution test of Sustained and Immediate release tablets. (**a**) Drug release from disc tablets with 100% infill and varying drug content, 5%, 10%, 20% and 30% *w*/*w*. (**b**) All tablet contained the same drug loading concentration of 10% *w*/*w* and varying infill, namely 17% (⌀_cell_ = 1.83 mm), 24% (⌀_cell_ = 1.22 mm), 45% (⌀_cell_ = 0.60 mm) and 100% (disc tablets). (**c**) Tablets with 45% infill and cell diameter of 0.60 mm tested for different drug loading concentrations.

Drug release profile varied depending on the tablet infill and drug concentration. Disc tablets (100% infill) were studied to test the impact of different drug concentrations on their drug release capabilities. The data in Figure 10 show that, while all formulations display sustained release over the period in consideration, the lower drug concentration samples (5% and 10%) displayed relatively faster drug release dissolution profiles compared to the higher concentration ones. With the XRD analysis it was found that higher drug loading inks contained a greater proportion of Fenofibrate in a crystalline form. The nature of Fenofibrate as a class II (BCS) drug, and the low solubility in its crystalline form, offers an explanation for this behaviour and the relatively slower release visible at higher concentrations. A similar conclusion was reached by Karolewicz and coworkers when studying the release behaviour of Fenofibrate–Pluronic F127 solid dispersions [50]. Specifically, they noted that as the polymer concentration increased the dissolution amount increased reaching its peak at 40% Fenofibrate content. As an explanation to this behaviour they suggested that the presence of the polymer caused the Fenofibrate to be found in a disaggregated state with an increased wettability. The discrepancy in the carrier concentration necessary to achieve the maximum drug release was attributed to the different hydrophilicity of the two materials. In particular, while Pluronic F127 is a hydrophilic material, Compritol HD5 contains a hydrophilic (PEG 8) and a hydrophobic component (behenic acid esters) which would explain the necessity of a higher concentration of carrier to obtain similar results.

When used to investigate the effect of surface area to volume (SA/V) ratio on drug release, disc tablets showed the slowest release, Figure 10b. In accordance with expectations [22,23,25,29], the rate of drug dissolution from the tablet tends to increase with the SA/V ratio and the smallest cell diameter (highest SA/V) belongs to the best performing tablet while the full disk is the worst in the sense of drug release rate. The disc tablet is indeed capable of releasing 87% of its drug content in 10 h. In contrast, the honeycomb tablet with the smallest cell diameter (0.60 mm) and highest SA/V was able to achieve the same result after only three hours and released more than 98% of the content by the end of the sixth hour. The geometries with cell diameters of 1.22 mm and 1.83 mm are in the region between the previous two curves, with the former releasing 90% by the sixth hour followed by the latter with an hour delay.

Notably when analysing the performance of the tablets with constant geometry (⌀_cell_ = 0.6 mm) and various drug loading, Figure 10c, the 5% *w*/*w* Fenofibrate displayed the relatively fastest drug release profile, with more than 92% of the initial drug released by the third hour and more than 98% by the fourth. The release profile of the 10% *w*/*w* tablet does not differ greatly from the 5% *w*/*w* delivering more than 98% of the total load in 6 h (and therefore approximately twice the amount of drug) showing that loading as well as geometry can be used as a control factor.

Multi-material tablets were utilised to assess the ability of a drug-free shell layer in delaying and controlling drug release from the core tablet.

**Figure 11:**
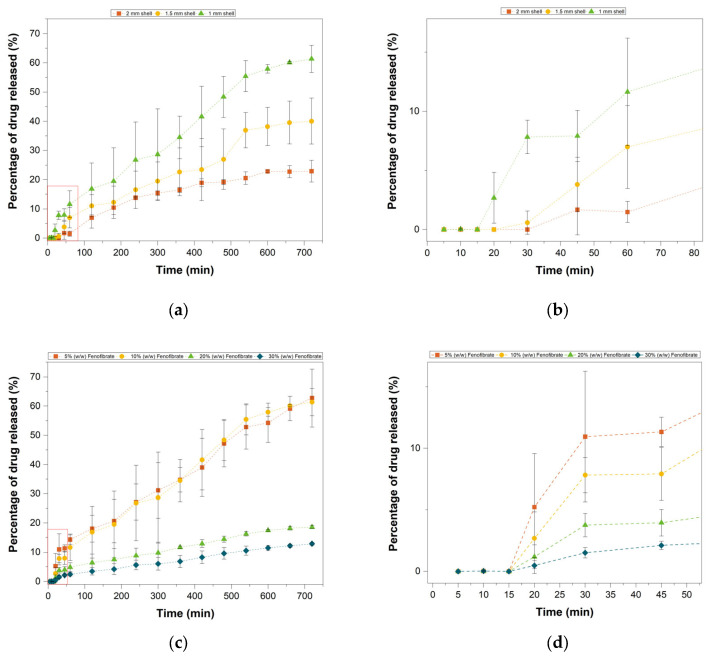
In vitro dissolution test of Delayed release tablets. (**a**) Tablets with single fixed core geometry and variation in shell thickness (1 mm, 1.5 mm and 2 mm). (**b**) Highlight of the initial delay in drug release. (**c**) Tablets with 1 mm shell and various drug concentration in the core (5%, 10%, 20% and 30% *w*/*w* Fenofibrate). (**d**) Highlight of the initial 15 min delay in drug release.

Samples with single fixed core geometry and shell thickness variation displayed a delayed release with a lag time of 15, 20, 30 min for 1 mm, 1.5 mm and 2 mm shell thicknesses, respectively (Figure 11a,b). After an initial delay the drug release was prompt and continued for the remaining 12 h. This is consistent with the literature [23,29,33], showing that the drug-free lipid shell induces a delay in the drug release and that shell thickness has a significant influence on dissolution profile. Notably, the thickness of the external layer not only prevents drug release for the initial delay time, but has a long-lasting effect on the dissolution rate, as it represents a diffusion barrier for drug release. From Figure 11a,b it is clear that the red curve (squares) corresponding to the 2 mm geometry shows a slower release profile compared to the green curve (triangles, 1 mm shell thickness) delivering just over 20% of the drug load in 12 h despite being delayed by only 15 min comparatively. From visual inspection of the tablets remains at the end of the experiment the tablets dissolved mainly by degradation and erosion.

As it displayed the fastest release profile, the thinner shell geometry was also tested with multiple Fenofibrate concentrations to ensure that the delayed behaviour was replicable at different drug loadings (Figure 11c,d). The tablets displayed the same delayed release of 15 min and afterwards developed the same dissolution pattern seen for both the disc and honeycomb tablets, where the fastest percentage drug release was achieved at the lowest drug loadings.

**Figure 12:**
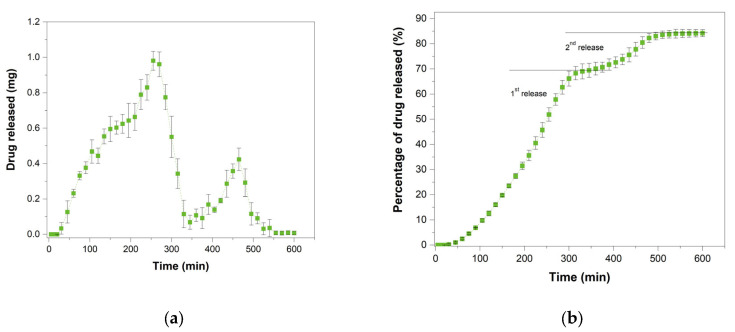
In vitro dissolution test of Pulsatile release tablets. (**a**) Shows the discrete release profile while (**b**) shows the cumulative release profiles obtained by addition of the data in the graph (**a**).

Lastly, a pulsatile release tablet design was used to test the depositional capabilities of the designed printer system by producing a tablet displaying complex geometry containing multiple nested layers of drug-loaded and drug free material to deliver a dual burst release. Due to the design restriction after an initial 20 min delay the drug was then released in an initial burst, during which 70% of the drug content was released within 330 min (5 h and 30 min) as visible in Figure 12, first release. This first pulse was then followed by a 45 min period of low-level release before the onset of a second significant release period lasting an additional 2 h second release phase (Figure 12). This behaviour is compatible with the assumptions and restrictions imposed on the design, highlighting the major influence of 3D geometry on drug delivery.

This last example furthermore demonstrates the capabilities of multi-material hot-melt ink-jet printing to produce solid dosage forms showing complex drug delivery profiles.

While an in vivo study will be necessary in the future for practical applications, Kadry and coworkers in their study of multi-materials medications with tailored drug release, similar to that proposed in this study, showed that the dissolution profiles achievable in vitro accurately simulated the in vivo drug-release profiles [36], suggesting that 3D tablets were capable of withstanding the harsher in vivo conditions and achieving the designed controlled drug release.

## 4. Conclusions

To conclude, this work demonstrates the capability of the designed hot-melt dual head 3D inkjet printer to deliver precise and accurate deposition of material droplets to produce multi-material solid dosage forms with complex geometries and drug distributions in a controlled manner. Concurrently, it was also shown that a commonly used pharmaceutical lipid, Compritol HD5 ATO, can be printed as single ink material, or in combination with a model drug, Fenofibrate, without the need for additional solvents, removing the need for time consuming and expensive post processing steps. In addition, the process is suitable to be used with any other drug or drugs with a higher degradation and lower melting point than the printing temperature (90 °C) and compatible with a lipidic carrier, widening the range of application. Based on Mao and co-workers’ work from 2016, we can assume that approximately 17% of all drugs (as per the collection published in Nature in 2007) have a melting point lower than 100 °C [51]. Most interestingly, when it comes to cardiovascular drugs, wildly present in poly-pill therapy, more than 20% of approved drugs fall in the range of interest. In addition, hot-melt inkjet printing is currently the only technique capable of using lipids as unique carrier. This is of particular interest, since the play a key role in the enhancement of the delivery of poorly soluble drugs.

All printed parts showed well-defined interior structures when observed via both optical and spectroscopic techniques, suggesting a high-level accuracy in material deposition with the drug homogeneously dis-tributed throughout the tablet (or desired areas within the tablet).

Furthermore, the capability of the system to fabricate printlets with tailored release profiles by smart designing the tablet geometry with drug-free and drug-laden material localisation was demonstrated. By tailoring the tablet infill, we were able to achieve sustained (100% infill) and immediate (45% infill) release.

For tablets with an external drug-free (barrier) layer after the initial delay the drug release was prompt and continued for the remaining 12 h. This is consistent with what can be found in the literature [23,29,33], showing that the use of a drug-free shell provides a delay in the drug release, and that shell thickness has a significant influence on dissolution profile.

Lastly, by introducing two concentric drug-loaded regions encapsulated in a drug-free matrix, we were able to obtain a dissolution profile showing two distinct burst releases.

The behaviours observed clearly demonstrate that a multi-material hot-melt ink-jet printer provides a suitable system to produce a complex three-dimensional drug delivery unit potentially applicable for the manufacture of personalised medicines. The 3D designs produced display a major and controllable influence on drug delivery, allowing us to tailor the drug release profiles in this system as desired (immediate, sustained, delayed and pulsatile release).

## Figures and Tables

**Table 1:** FTIR characterisation of tailored release tablets via the intensity of the Fenofibrate characteristic 765 cm^−1^ peak.

	Geometry [mm]	Fenofibrate (*w*/*w*)	Tablet [a.u.]	Reference [a.u.]	Percentage Error [%]
Immediate	Channel				
I^2^_a_	600	10%	38.79 ± 1.18	37.01 ± 3.48	4.6
I^2^_b_	1220	10%	38.62 ± 0.70	37.01 ± 3.48	4.2
I^2^_c_	1830	10%	39.63 ± 2.44	37.01 ± 3.48	6.6
I^1^_a_	600	5%	24.01 ± 1.18	23.63 ± 2.82	1.6
I^2^_a_	600	10%	38.79 ± 1.18	31.01 ± 3.48	20.1
I^3^_a_	600	20%	43.01 ± 2.44	42.47 ± 2.93	1.3
I^4^_a_	600	30%	70.16 ± 2.44	70.48 ± 0.12	0.5
Delayed	Shell				
D^2^_a_	1	10%	36.81 ± 1.83	37.01 ± 3.48	0.5
D^2^_b_	1.6	10%	37.57 ± 2.00	37.01 ± 3.48	1.5
D^2^_c_	2	10%	37.85 ± 1.59	37.01 ± 3.48	2.2
D^1^_a_	1	5%	23.56 ± 1.87	23.63 ± 2.82	0.3
D^2^_a_	1	10%	36.81 ± 1.83	31.01 ± 3.48	15.8
D^3^_a_	1	20%	43.12 ± 1.13	42.47 ± 2.93	1.5
D^4^_a_	1	30%	70.47 ± 0.60	70.48 ± 0.12	0.01
Pulsatile					
Core		10%	37.95 ± 2.04	37.01 ± 3.48	2.5
Ring		10%	37.50 ± 2.40	37.01 ± 3.48	1.3

Note: when identifying a sample, the capital letters specify the type of release profile (I = immediate, D = delay, P = pulsatile). The subscript letter indicates different geometries within a set and the appendix refers to the drug concentration (%) in the ink used to print the drug loaded regions.

## Data Availability

Data are available upon request or at the Nottingham Research Data Management Repository following the link: https://rdmc.nottingham.ac.uk/, accessed on 21 August 2021.
